# Altered cortical thickness, degree centrality, and functional connectivity in middle-age type 2 diabetes mellitus

**DOI:** 10.3389/fneur.2022.939318

**Published:** 2022-11-04

**Authors:** Shangyu Kang, Yuna Chen, Jinjian Wu, Yi Liang, Yawen Rao, Xiaomei Yue, Wenjiao Lyu, Yifan Li, Xin Tan, Haoming Huang, Shijun Qiu

**Affiliations:** ^1^The First School of Clinical Medicine, Guangzhou University of Chinese Medicine, Guangzhou, China; ^2^Department of Endocrinology, The First Affiliated Hospital of Guangzhou University of Chinese Medicine, Guangzhou, China; ^3^Department of Radiology, The First Affiliated Hospital of Guangzhou University of Chinese Medicine, Guangzhou, China

**Keywords:** type 2 diabetes mellitus, brain, middle age, resting-state fMRI, surface-based morphometry

## Abstract

**Purpose:**

This study aimed to investigate the changes in brain structure and function in middle-aged patients with type 2 diabetes mellitus (T2DM) using morphometry and blood oxygen level-dependent functional magnetic resonance imaging (BOLD-fMRI).

**Methods:**

A total of 44 middle-aged patients with T2DM and 45 matched healthy controls (HCs) were recruited. Surface-based morphometry (SBM) was used to evaluate the changes in brain morphology. Degree centrality (DC) and functional connectivity (FC) were used to evaluate the changes in brain function.

**Results:**

Compared with HCs, middle-aged patients with T2DM exhibited cortical thickness reductions in the left pars opercularis, left transverse temporal, and right superior temporal gyri. Decreased DC values were observed in the cuneus and precuneus in T2DM. Hub-based FC analysis of these regions revealed lower connectivity in the bilateral hippocampus and parahippocampal gyrus, left precuneus, as well as left frontal sup.

**Conclusion:**

Cortical thickness, degree centrality, as well as functional connectivity were found to have significant changes in middle-aged patients with T2DM. Our observations provide potential evidence from neuroimaging for analysis to examine diabetes-related brain damage.

## Introduction

Type 2 diabetes mellitus (T2DM) accounts for more than 90% of patients with diabetes. At present, the incidence and prevalence of T2DM continue to rise globally (1). Patients with T2DM have been reported to have a higher incidence of cognitive decline and Alzheimer's Disease (AD) (2–4). T2DM may be associated with an accelerated decrease in the speed of processing, executive function, and the loss of memory (5). Despite a consensus on the effect of T2DM on brain atrophy in this stage, the association of diabetes-related brain damage is less well-characterized. Aging is one of the strongest risk factors for brain atrophy (6). Elderly patients with T2DM were examined in most studies (7, 8), while most of them had developed atrophic brains and cognitive impairment, and effective interventions are limited at this stage. Therefore, early attention needs to be paid to middle-aged patients with T2DM before irreversible brain damage happens (5, 9, 10).

Magnetic resonance imaging (MRI) is a non-invasive technique to study central nervous system alterations. Meanwhile, a recent longitudinal study suggested that brain atrophy might have occurred in middle-aged T2DM patients (11). The clinical manifestation of brain atrophy in middle age is not as obvious as that in old age. Studies showed small changes in brain structure in obese adolescents (12, 13). However, there is still lacking evidence of brain structural and functional alterations in middle-aged T2DM patients. It could be of great clinical implication to prevent and mitigate the progress of diabetes-related brain damage that changes in the brain can be found in middle-aged patients with T2DM, and this kind of neurodegenerative alterations, linked with age, cognitive performance, and pathogenic changes, can be readily studied using cortical morphological and functional analysis.

Previous studies showed that T2DM patients had undergone varying degrees of changes in the structure of the brain (14–16). Neurodegeneration alterations can be studied using cortical morphological analysis. Several studies used surface-based morphometry (SBM) to assess changes in cortical thickness in AD (17, 18). Simultaneously, several studies used voxel-based morphometry (VBM) and found that gray matter (GM) volume is reduced in patients with T2DM (19, 20), suggesting that GM changes might be a potential biomarker for brain damage in T2DM early evaluation. SBM is a more advancing technique illustrating the morphological cortical/GM changes compared to VBM (21). Only a few SBM studies were conducted on T2DM (22), especially in the middle-aged group. Resting-state functional magnetic resonance imaging (rs-fMRI) and brain network theory have been widely used to understand the neuropathophysiology of diabetes-related brain damage (23). Different changes were found in various functional imaging indexes (24–26). However, the aforementioned studies mainly aimed at elderly patients with T2DM. Because the damage of hyperglycemia to the nervous system is a chronic and lasting process (27, 28), identifying changes in middle-aged patients with T2DM could lead to a better understanding of diabetes-related brain damage.

The purpose of this study was to identify diabetes-related modulations of cortical thickness and neural activity in middle-aged patients, to reveal diabetes-related brain damage in neuroimaging. In this study, we used SBM to detect cortical thickness in middle-aged patients with T2DM. Subsequently, we used rs-fMRI to reveal spontaneous or internal connections within and between regions of the brain to further understand the brain network of middle-aged patients with T2DM.

## Materials and methods

### Participants

This study was recognized by the ethics committee of the Guangzhou University of Traditional Chinese Medicine. Each subject is demanded to sign a medical consent form for medical procedures. A total of 44 patients with T2DM were recruited from July 2019 to October 2021, who met the diagnostic criteria of T2DM published by the American Diabetes Association in 2014 and were all right-handed. Middle-aged people were defined as people between the ages of 40 and 60 years. At the same time, the neurocognitive function of all patients with T2DM was assessed based on the Montreal Cognitive function Assessment (MoCA) scale and Mini-mental State Examination (MMSE) scores. The following characteristics were used to eliminate participants: other diabetes, organic brain injury (such as brain trauma, cerebrovascular disease, or tumor), mental health problems, systemic diseases, drug abuse, and a history of alcohol and tobacco addiction. During the same period, 45 HCs matched for age, gender, and education were recruited. The inclusion criteria were as follows: glycosylated hemoglobin <6.0% and fasting blood glucose <7.0 mmol/L. Considering the low incidence of cognitive impairment in the middle-aged group (29), we excluded HCs whose MoCA and MMSE scores were both <26. Other exclusion criteria were the same as for the T2DM group.

### Clinical measurements and cognitive testing

Without shoes or heavy clothing, the body weight and height were measured. The body mass index (BMI) was computed by dividing the weight in kilos by the squared height in meters. In a sitting position, blood pressure was recorded two times at a 5-min intervals on the left arm. Glycosylated hemoglobin, fasting blood glucose (FBG), and fasting insulin (FINS) were among the clinical biochemical indicators in individuals with T2DM. HOMA-IR was calculated using the method HOMA-IR = FBG × FINS/22.5. The duration of T2DM was estimated based on the patient's self-reported initial time.

All individuals were required to complete a series of neuropsychological tests, including MoCA (30), MMSE (31), Grooved Pegboard Test (GPT) (32), Auditory Verbal Learning Test (AVLT) (33), and Digit Symbol Substitution Test (DSST) (34).

### MRI acquisition

A 3.0T Siemens MAGNETOM Prisma clinical MRI scanner with a 64-channel head coil was used to collect MRI data. During MRI image acquisition, the participants closed their eyes and stayed awake. Cushions are used to decrease head movement and earplugs are used to lessen noise impact. To rule out organic lesions in the brain (cerebral infarction, hemorrhage, trauma, and space-occupying lesions), all participants acquired T2WI and T2-FLAIR sequences.

The echo plane imaging sequence parameters were as follows: repetition time (TR) = 500 ms; echo time (TE) = 30 ms; field of view (FOV) = 244 mm × 244 mm; slices = 35; thickness = 3.5 mm; and voxel size = 3.5 mm × 3.5 mm × 3.5 mm; 960 volumes were transversely acquired. Structural images were obtained using magnetization-prepared rapid gradient echo sequences: TR = 2530 ms; TE = 2.98 ms; inversion time (TI) = 1100 ms; flip angle = 7°; FOV = 224 mm × 256 mm; slices =192; thickness = 1 mm; and voxel size = 1 mm × 1 mm × 1 mm.

### Image processing and analysis

#### SBM analysis

The Computational anatomy toolbox (CAT12) (http://www.neuro.uni-jena.de/cat/) was used to conduct the SBM on the MATLAB 2016b platform. The projection-based thickness method was used to estimate the cortical thickness and central surface of the left and right hemispheres (35). First, we reviewed and converted the raw DICOM scans into the Neuroimaging Informatics Technology Initiative (NIFTI) format (36). The processing pipeline was as follows: Took each participant's brain images segmentation into GM, white matter, and cerebrospinal fluid; and affine registration to MNI template space, which involved diffeomorphic anatomical registration using Diffeomorphic Anatomical Registration Through Exponentiated Lie algebra (DARTEL) to a 1.5-mm isotropic adult template and subsequent nonlinear deformation. For subsequent cortical thickness analysis, we chose the option: “Surface and thickness estimation.” The cortical thickness was calculated for each participant with default parameter settings. To check for homogeneity of the images of the cerebral cortex characteristics, we assessed the homogeneity of SBM data in the “Check data quality” section in CAT12. High correlation values mean that data are more similar to each other. All of the images exhibited a good correlation (>0.85). Finally, a Gaussian kernel with a full-width-half-maximum (FWHM) of 15 mm was used to smooth the images for cortical thickness.

#### Preprocessing of resting-state fMRI data

Statistical Parametric Mapping (SPM12) (https://www.fil.ion.ucl.ac.uk/spm/) and Resting-State fMRI Data Analysis Toolkit plus V1.2 (RESTplus V1.2) (http://www.restfmri.net/forum/restplus) were used to preprocess the images on the MATLAB 2013b platform. To find the volume in steady-state magnetization, the first 10 time points were eliminated. The remaining 950 images had the head motion adjusted. Additionally, conducting slice timing during preprocessing was not necessary for images with lower-echo time (37). Excessive head motion was defined as more than 2 mm translation or more than 2° rotation in either direction. To ensure a more precise spatial normalization, the 3D-T1WI was utilized to guide rs-fMRI registration *via* the unified segment and DARTEL method. We used the Friston 24-parameter head motion (HM) model, which included 6 HM parameters, 6 HM parameters one-time point before, and the 12 corresponding square items to regress out the head motion effects from realignment (38). To decrease noise, the data were filtered to a 0.01–0.08 Hz range.

#### Degree centrality and functional connectivity analysis

Degree centrality (DC) parameters were calculated using RESTplus. The algorithm for DC has been reported previously (39) and can be summarized as follows. First, the time series were extracted from the preprocessed resting-state fMRI data to calculate a correlation matrix using the temporal Pearson's correlation of the time series between certain voxels. Then, fully connected binary graphs were built with a threshold of correlation r = 0.25; the selection of this parameter was consistent with our previous study (20). When the correlation between two voxels was greater than the threshold, the binary graph was 1, otherwise, it was 0. According to the adjacency matrix of the graph, the DC parameters were calculated for each voxel by the addition of the correlations of each voxel. The values in each voxel were transformed to z-values using the Fisher z-transformation to improve normality. Then, we used RESTplus to smooth DC values as a 6-mm (FWHM) Gaussian kernel.

The peak point in the results of the DC analysis was defined as the coordinates of the seed area, in which the radius was set to 6 mm. After defining the seed area, we performed functional connectivity (FC) analysis based on the seed area. We computed Pearson's correlation coefficients between the seed area and the remaining brain voxels. Then, a Fisher r-to-z transformation was displayed to improve normality. Finally, we obtained z-FC maps of each individual for further analysis.

### Statistical analyses

#### Demographic and clinical data

These statistical analyses were performed using the SPSS software package (version 26.0). First, the Kolmogorov–Smirnov test was used to determine whether the data were normal. The two-sample *t*-test was used to assess the normalized data. For non-normalized data, we used the Mann–Whitney nonparametric tests. Gender and other categorical variables were utilized to find differences using the Chi-Squared test. *P* < 0.05 was used as the statistical significance level.

#### Cortical thickness

The analyses of cortical thickness imaging were compared between the patients with T2DM and HCs using two-sample *t*-tests in CAT12 with age, gender, and education as covariates. Family-wise error (FWE) correction was performed to correct for multiple comparisons; *P* < 0.05 was considered statistically significant. Then, we reported the surviving clusters in the Desikan–Killiany (DK40) atlas (40), which presented an atlas for subdividing the human cerebral cortex into standard gyral-based neuroanatomical regions.

#### Degree centrality and functional connectivity

To analyze DC and FC differences, we performed a two-sample *t*-test between the two groups in the SPM12. Age, gender, education, and head movement parameters were set as covariates. To correct the DC result for multiple comparisons, we used the Gaussian random field (GRF) (two-tailed, voxel-level *P* < 0.005, cluster-level *P* < 0.05) method in the RESTplus. The corrected cluster size threshold was 95 voxels.

The analysis of FC was also performed in the SPM12 statistical module. The statistical methods used were consistent with DC. To improve the credibility of FC results, the result was presented at the statistical threshold of *P* < 0.01 using voxel-level FWE correction.

## Results

### Demographic, clinical, and cognitive characteristics

Table 1 shows the demographic, clinical, and cognitive data of all the subjects. In terms of age, gender, and education level, there was no statistically significant difference between the two groups.

**Table 1:** Demographic and clinical data of all participants.

	**T2DM (*n =* 44)**	**HCs (*n =* 45)**	**Statistics**	***P* value**
**General and clinical data**
Age (years)	50.43 ± 5.12	49.93 ± 5.85	*t =* 0.427	0.670
Gender (male/female)	26/18	27/18	*χ^2^* = 0.008	0.930
Education (years)	12 (9, 15)	12 (9, 12)	*z* = −0.543	0.587
Duration (years)	4.5 (2, 6.75)	N/A	N/A	N/A
HbA1c (%)	8.25 (6.9, 10.8)	N/A	N/A	N/A
FBG (mmol/L)	7.56 (5.92, 9.16)	N/A	N/A	N/A
FINS (μIU/ml)	6.83 (4.61, 15.57)	N/A	N/A	N/A
HOMR-IR	2.57 (1.64, 5.54)	N/A	N/A	N/A
BMI (kg/m^2^)	24.15 ± 3.29	23.36 ± 2.80	*t =* 1.229	0.222
SBP (mmHg)	126.93 ± 11.00	125.91 ± 13.35	*t =* 0.393	0.695
DBP (mmHg)	83.09 ± 8.36	82.56 ± 6.25	*t =* 0.343	0.733
**Cognitive tests**
MoCA score	27 (25, 29)	27 (26, 29)	*z* = −0.249	0.803
MMSE score	28.97 (27.4, 29)	28 (27, 29)	*z* = −0.790	0.430
GPT (R)	73 (65, 82.75)	70 (61, 82)	*z* = −0.813	0.416
GPT (L)	79.56 (73.25, 89.75)	82 (73, 95)	*z* = −0.920	0.358
AVLT (immediate)	24 (20, 27.75)	23 (19, 26)	*z* = −1.016	0.310
AVLT (5 min)	10 (8, 11)	10 (8, 10)	*z* = −0.941	0.346
AVLT (20 min)	9 (7, 11)	9 (8, 11)	*z* = −0.125	0.901
DSST	46.91 ± 11.07	44.58 ± 14.34	*t =* 0.859	0.393

Two-sample t-tests for normalized data, Mann–Whitney tests for non-normalized data, and Chi-Squared tests for gender. N/A, not applicable.

T2DM, type 2 diabetes mellitus; HCs, healthy controls; HbA1c, glycosylated hemoglobin; FBG, fasting blood glucose; FINS, fasting insulin; BMI, body mass index; SBP, systolic blood pressure; DBP, diastolic blood pressure; MoCA, Montreal Cognitive function Assessment; MMSE, Mini-mental State Examination; GPT, Grooved Pegboard Test; AVLT, Auditory Verbal Learning Test; DSST, Digit Symbol Substitution Test.

There were no significant differences in the MoCA, MMSE, GPT (R), GPT (L), AVLT (immediate), AVLT (5 min), AVLT (20 min), and DSST scores between the two groups on the cognitive exam. Table 1 summarizes the results of the cognitive tests.

### Cortical thickness

**Figure 1:**
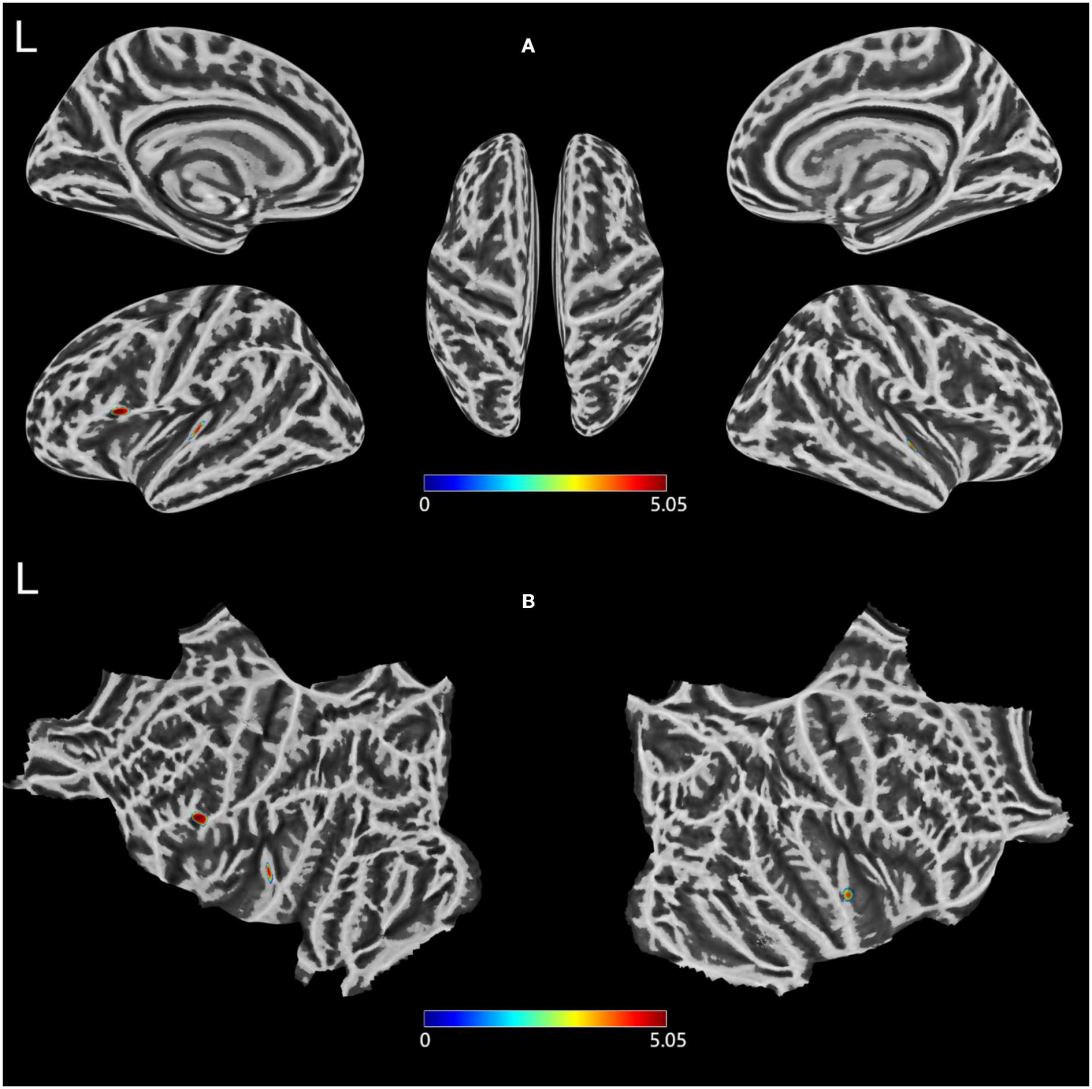
**(A,B)** Clusters showing significantly changed Cortical thickness in the T2DM group compared to the HCs group. Two-sample *t*-test, FWE corrected *P* < 0.05. T2DM, Type 2 diabetes mellitus; HCs, healthy controls; FWE, Family-wise error; L, left.

Compared with HCs, patients with T2DM exhibited thinner cortical thickness, including the left pars opercularis and left transverse temporal and right superior temporal gyri (Figure 1; Table 2).

**Table 2:** Comparison of cortical thickness between T2DM and HCs groups.

	**Atlas regions**	**Peak MNI**			**Cluster-size**	**T**
L	Pars opercularis	−53	15	8	305	−5.04
	Transverse temporal	−48	−21	8	184	−4.87
R	Superior temporal	51	−11	−2	111	−4.76

T2DM, type 2 diabetes mellitus; HCs, healthy controls; MNI, Montreal Neurological Institute; L, left; R, right.

### Degree centrality

**Figure 2:**
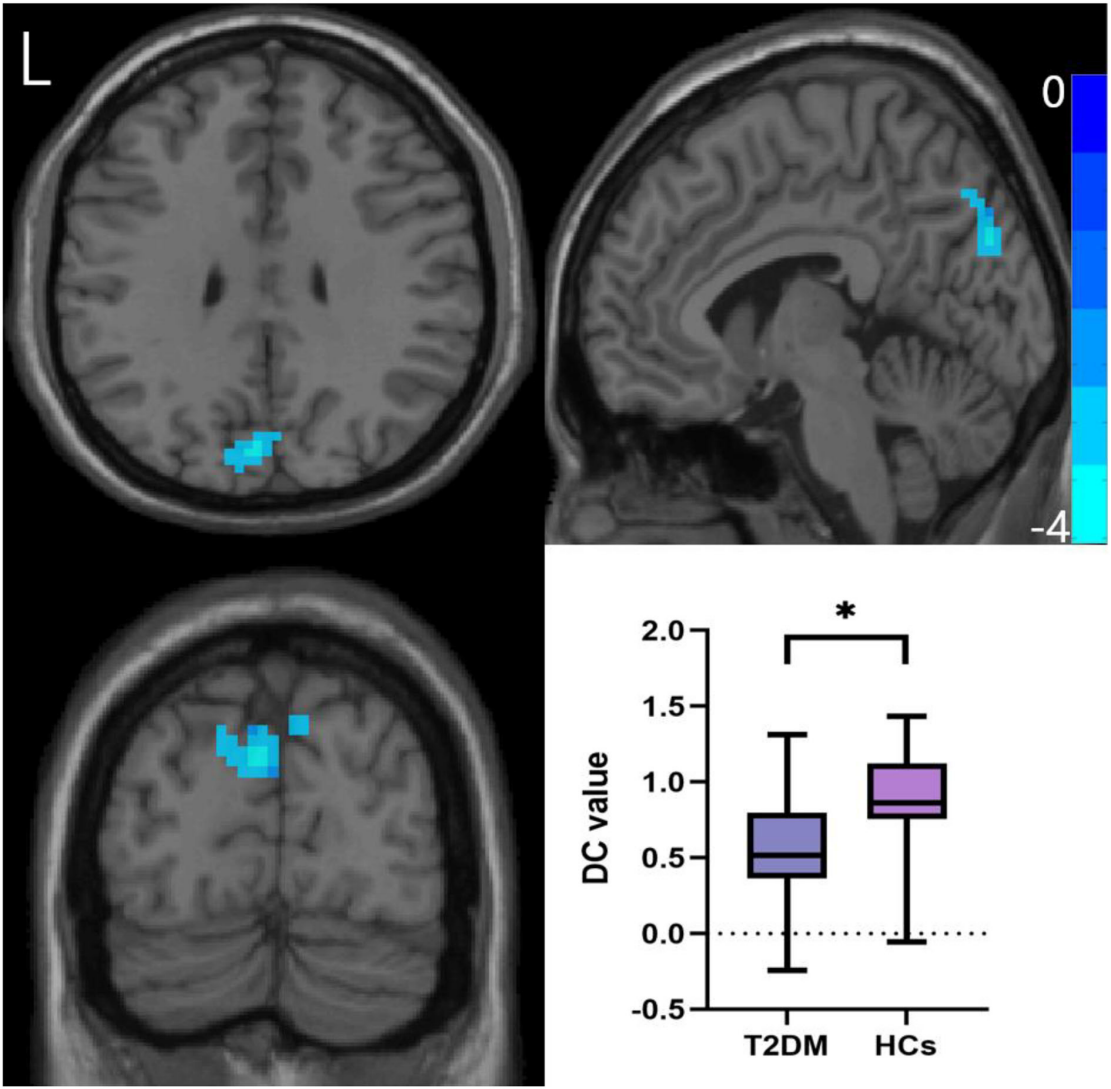
Comparison of DC values between the two groups. Two-sample *t*-test, Gaussian random field correction (two-tailed, voxel-level *P* < 0.005, cluster-level *P* < 0.05). DC, degree centrality; L, left. *P < 0.05.

After GRF multiple comparison correction, only one cluster in the T2DM group with decreased DC survived after intergroup comparison (Figure 2; Table 3), involving the cuneus and precuneus, compared with HCs.

**Table 3:** Comparison of DC and FC between T2DM and HCs groups.

	**Atlas regions**	**Peak MNI**			**Number of voxels**	**T**
**DC**
R	Precuneus Cuneus	6	−66	51	112	−4.089
**FC**
R	ParaHippocampal Hippocampus	22	−33	−9	201	−7.877
L	ParaHippocampal Hippocampus	−27	−33	−6	173	−7.707
L	Precuneus	−9	−57	51	35	−7.356
L	Frontal Sup	−18	−3	63	54	−6.566

T2DM, type 2 diabetes mellitus; HCs, healthy controls; DC, Degree Centrality; FC, Functional Connectivity; MNI, Montreal Neurological Institute; L, left; R, right.

### Functional connectivity

**Figure 3:**
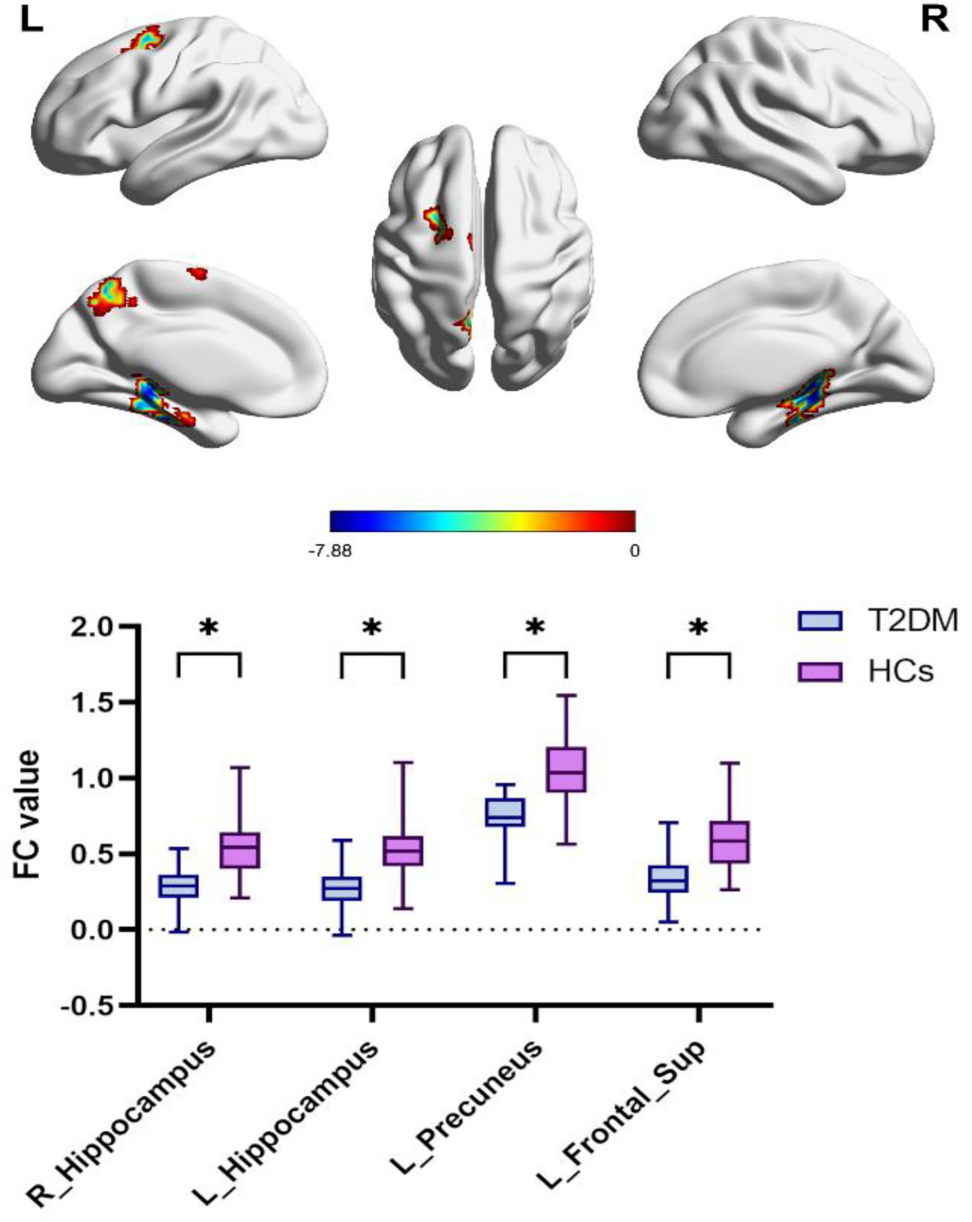
Differences in precuneus-based functional connectivity between the T2DM group and HCs group. Two-sample *t*-test, FWE corrected *P* < 0.01. T2DM, Type 2 diabetes mellitus; HCs, healthy controls; FWE, Family-wise error; L, left; R, right. *P < 0.05.

The right parahippocampal, right hippocampal, left fusiform, left parahippocampal, left hippocampus, left precuneus, and left frontal sup areas were shown to have decreased FC in the T2DM group, according to the hub-based FC analysis (Figure 3; Table 3).

## Discussion

According to this study, the best of our knowledge is focused on the morphological indicator of cortical thickness. We found that middle-aged patients with T2DM have reduced cortical thickness of the left pars opercularis, left transverse temporal, and right superior temporal gyri compared with HCs. Cortical thickness partly characterizes the development of brain tissue and is associated with diseases such as neurodegeneration. In addition, the version of understanding is that T2DM accelerates the aging of the brain, and this cortical aging accelerates the transformation of cognitive impairment (41–43). A predictive change that accelerated cortical thinning is a feature of cognitive impairment and dementia may have occurred before clinical diagnosis (44). Despite no significant difference in cognitive testing between middle-aged patients with T2DM and HCs, significant changes were found in cortical thickness, indicating that the change in brain structure was a more sensitive biological marker that could provide more powerful evidence for brain damage caused by T2DM.

A previous study of T2DM in the elderly found that long-term adiposity might have a detrimental impact on the volume of brain regions (45). The results included the area of pars opercularis, which is the part of the inferior frontal gyrus that overlies the insular cortex (46). The results of resting-state fMRI indicated that the enhanced functional connectivity of the left hippocampus with the left inferior frontal gyrus significantly correlated with disease severity. Our study found that the cortical thickness of the left pars opercularis in middle-aged T2DM decreased significantly, which might be an important cause of accelerated brain atrophy.

Meanwhile, compared with HCs, middle-aged T2DM patients showed significantly reduced cortical thickness in the left transverse temporal gyrus. The transverse temporal gyrus plays an important role in auditory processing (47). Previous research reported cortical thickness reductions in the temporal gyrus in patients with T2DM (48). The findings indicated that T2DM probably caused brain damage in specific regions. Graph theory analysis suggested that the brain function network of patients with T2DM has changed in the temporal lobe (49). The results provided a theoretical basis for diabetic pathophysiology and clinical presentation. To sum up, the evidence suggested that morphology changes in the temporal lobe of middle-aged T2DM may link to diabetes-related brain damage.

The right superior temporal gyrus is mainly responsible for processing objects and spatial information (50). A meta-analysis confirmed that the right superior temporal gyri had resting-state function alterations in T2DM relative to healthy people (51). VBM analysis found that gray matter volume in patients with T2DM was decreased in the right superior temporal gyrus (52). However, the relationship between the structural and functional changes of the right superior temporal gyrus is not clear, emphasizing the need for further research.

Our study also found that in the cuneus and precuneus, the DC value of brain function in T2DM decreased. DC is a local (direct) network connectivity of indicator, which shows that the higher-order cortical correlation regions are important (39). Precuneus is an important part of the default mode network (DMN). DMN plays an important role in primary perceptual control and advanced cognitive processing, in which the precuneus is the core node of DMN (53). Similar alteration patterns in the brain can be observed in T2DM patients, in which the population at risk for AD show altered brain activity in the DMN before cognitive dysfunction (54). Another study of neurological changes in patients with T2DM found reduced brain activity in the left precuneus (55). Moreover, it is reported that memory impairment in patients with early AD could be improved with stimulation to the precuneus in AD subjects by high-frequency repetitive transcranial magnetic stimulation (56). Together with the observations of the decrement DC value in cuneus and precuneus regions among middle-aged patients with T2DM, the results emphasized the importance of cuneus and precuneus in fundamental cognitive functioning and the DMN architecture of T2DM and functional changes in those regions may serve as sensitive imaging biomarkers for early monitoring of the progress of brain damage in T2DM.

Subsequently, we found, in the FC analysis based on the precuneus as the ROI, a decreased functional connection between the anterior cuneate lobe and the bilateral hippocampus and parahippocampal gyrus in T2DM. Previous studies showed that hippocampal damage was related to the severity of diabetes and cognitive impairment (57–61). Some studies used the hippocampus as the seed region (59, 60, 62) and found that the FC between the hippocampus and DMN was weak, which was related to memory and cognitive ability. Another study segmented hippocampal subregions and found that several hippocampal subfield volumes were significantly associated with memory scores, highlighting the key role of the hippocampus in memory decline (63). In T2DM subjects included in the present study, hippocampal functional declines were already initiated despite no cognitive impair manifestations. The hippocampus is an important structure for proper functional cognition. However, most studies were cross-sectionally designed, and those continuous alterations are inaccessible. Long-term modification in the hippocampus should be an important topic in future investigations.

We also found a decrease in the functional connection between the precuneus and the left superior frontal gyrus in T2DM. Few studies were conducted on the left frontal sup in T2DM. One study on diabetic retinopathy reported a decrease in the DC value of the right superior frontal gyrus (64). A study of transcranial direct current stimulation (tDCS) in treating MCI found (65) that the intensity and synchronization of bilateral frontal lobe activity improved after tDCS. Nonetheless, evidence is still limited on the left superior frontal gyrus aberrant among T2DM. Therefore, further studies can be performed on the frontal lobe in T2DM to enhance the understanding of diabetes-related brain damage.

Several limitations also need to be illustrated in this study. First, we use a relatively small sample size. Hence, a sample size of more patients qualified for this study is urgently needed. Second, causal relationships or structural progression and functional changes cannot be shown in the cross-sectional design. Therefore, the longitudinal design should be used in future studies. Third, our results should be comprehended cautiously. Due to the strict threshold, only a few regions are analyzed in this manuscript. Multimodal MRI can better demonstrate the reliability of the results and requires further exploration.

## Conclusion

Cortical thickness, DC, and FC levels were shown to be reduced in middle-aged patients with T2DM in our preliminary study. These parameters could potentially serve as biomarkers for predicting brain atrophy progression. Especially, we found that SBM could be able to provide more information about the diversification of cortical morphology in T2DM. To summarize, our findings in the neuroimaging field suggested that middle-aged patients with T2DM had undergone abnormal changes in brain structure and function. The cortical thickness, DC, and FC might serve as promising indicators to reflect and enhance our understanding of diabetes-related brain damage.

## Data availability statement

The raw data supporting the conclusions of this article will be made available by the authors, without undue reservation.

## Ethics statement

The studies involving human participants were reviewed and approved by Guangzhou University of Traditional Chinese Medicine. The patients/participants provided their written informed consent to participate in this study.

## Author contributions

JW, XT, YLia, WL, YLi, YR, and XY contributed to the design of the study. SK, JW, XY, and HH performed the data processing. XT, HH, and SQ provided theoretical guidance. SK performed the data analysis. SK and YC drafted the manuscript. All authors revised the manuscript and approved the submitted version.

## Funding

This research was funded by the Key International Cooperation Project of the National Natural Science Foundation of China (81920108019) and the Medical Scientific Research Foundation of Guangdong Province (A2021182).

## Conflict of interest

The authors declare that the research was conducted in the absence of any commercial or financial relationships that could be construed as a potential conflict of interest.

## Publisher's note

All claims expressed in this article are solely those of the authors and do not necessarily represent those of their affiliated organizations, or those of the publisher, the editors and the reviewers. Any product that may be evaluated in this article, or claim that may be made by its manufacturer, is not guaranteed or endorsed by the publisher.

## Abbreviations

AVLT: Auditory Verbal Learning Test
BMI: body mass index
DC: degree centrality
DSST: Digit Symbol Substitution Test
FC: functional connectivity
FBG: fasting blood glucose
FINS: fasting insulin
FOV: field of view
FWE: Family-wise error;Grooved Pegboard Test
GM: gray matter
GRF: Gaussian random field
HCs: healthy controls
MMSE: Mini-Mental State Examination
MNI: Montreal Neurological Institute
MoCA: Montreal Cognitive function Assessment
SBM: Surface-based morphometry
T2DM: Type 2 diabetes mellitus
TMT: Trail Making Test
TR: repetition time
TE: echo time.
